# Opportunities to improve the adoption of health-related quality of life evidence as part of the French Health Technology Assessment process

**DOI:** 10.1186/s12961-023-01081-8

**Published:** 2023-12-19

**Authors:** Hugo Larose, Myrto Lee, Jens Grueger, Amélie Anota, Nicolas Naïditch, Bruno Falissard, Mario Di Palma, Olivier Chassany, Laura Khalfallah-Neelz, Sarah Palazuelos-Muñoz, Aymeric Tetafort

**Affiliations:** 1 The Boston Consulting Group, 80 Charlotte Street, London, W1T 4QS United Kingdom; 2 University of Washington, Seattle, WA United States of America; 3 The Boston Consulting Group, Zurich, Switzerland; 4 Department of Clinical Research and Innovation and Department of Human and Social Sciences, Centre Léon Bérard, Lyon, France; 5 French Federation of Diabetics, Paris, France; 6 CESP, Université Paris-Saclay, APHP, Villejuif, France; 7 Gustave Roussy Cancer Center, Paris-Saclay University, Villejuif, France; 8 Health Economics Clinical Trial Unit (URC-ECO), Hôpital Hotel-Dieu, AP-HP, Paris, France; 9 Patient-Reported Outcomes Research Unit (PROQOL), Université Paris Cité, Paris, France; 10 AstraZeneca, Paris, France

**Keywords:** Health-related quality of life (HRQoL), Health technology assessment (HTA), Haute Authorite de la Sante (HAS), EUnetHTA, Patient perception, Testing hierarchy, Trial design, Data collection

## Abstract

**Objectives:**

Patient’s health-related quality of life (HRQoL) is an important outcome measure that is considered by many payers and health technology assessment (HTA) bodies in the evaluation of treatments. We aimed to identify opportunities for HRQoL to be further incorporated into the assessment of the French HTA by comparing three health systems. We put forward recommendations that could bring further innovations to French patients.

**Methods:**

We reviewed methodologies by the French, German and British HTA, and conducted a systematic review of all French (*n* = 312) and German (*n* = 175) HTA appraisals from 01 January 2019 to 31 December 2021. We also setup an advisory board of 11 ex-HTA leaders, payers, methodologists, healthcare providers and patient advocates, from France, Britain and Germany, to discuss opportunities to improve acceptance and adoption of HRQoL evidence in France.

**Results:**

Our systematic review of HTA appraisals showed a higher HRQoL data rejection rate in France: in > 75% of cases the HRQoL evidence submitted was not accepted for the assessment (usually for methodological reasons, for example, data being considered exploratory; 16–75% of the appraisals mentioned HRQoL evidence, varying by therapeutic area). Overall, we found the French HTA to be more restrictive in its approach than IQWiG.

**Conclusions:**

Based on these findings we articulate collaborative proposals for industry and the HAS to improve acceptance of HRQoL evidence and create a positive feedback loop between HAS and industry along four dimensions (1) patient perception, (2) testing hierarchy, (3) trial design and (4) data collection.

**Supplementary Information:**

The online version contains supplementary material available at 10.1186/s12961-023-01081-8.

## Background

Health technology assessment (HTA) bodies and payers are increasingly focused on patient-relevant end-points and the patient voice to ensure that patients benefit from an improved health-related quality of life (HRQoL) and are able to access these innovations rapidly. Patient-relevant end-points are also essential to capture the patient experience on treatment with generation of evidence to inform the clinical benefit–risk assessment. The value assessment put forward by The Professional Society for Health Economics and Outcomes Research (ISPOR) [1], American Society of Clinical Oncology (ASCO) [2], European Society for Medical Oncology (ESMO) [3] and others increasingly focus on assessing new products as holistically as possible, including the impact products have on patients’ quality of life. HRQoL measures are not new; they have been piloted since the 1990s [4]. These measures are continuously adjusted to enable relevance to patients and can be captured reliably in an efficient way. While some payers/HTA prefer generic scales, others, such as Institute for Quality and Efficiency in Health Care (IQWiG), in Germany, traditionally prefer disease-specific scales [5]. Other HTA yet, such as the National Institute for Care Excellence (NICE) in the United Kingdom, use a wholly different system, by looking at utility values, usually derived from instruments such as EuroQol five-dimensions (EQ-5D) generic questionnaire [6, 7].

Taking the impact on patient quality of life is increasingly important: for example, in oncology, for late-stage or metastatic disease, treatments may last for a long period of time, meaning that patients need to be able to, and learn to, live with the side-effects of the treatment trialed. The impact on quality of life therefore becomes a significant part of the patient’s life for weeks and months, and can therefore no longer be ignored.

In France, the question of how to best assess impact of new therapies on patient HRQoL is very topical and gaining prominence: the Haute Authorité de la Santé (HAS), the French HTA, has recently published a number of reports on this topic. These range from reports on international use of/guidelines on patient-reported outcome and experience measures (PROMs and PREMs) [8, 9] to guidelines on how to collect HRQoL data as part of real-world evidence studies [10], including in the context of the new early access program [11, 12]. The 10-year national strategy for fighting cancer specifically mentions improving quality of life as one of its foundational aims [13] and the High Council on Public Health recently published a report that underlines both the importance of capturing and assessing HRQoL as well as the complexity of integrating this into the HTA assessment [14].

We aimed to identify the evidence needs and potential opportunities in how HRQoL could be further incorporated into the assessment of the HAS by comparing HTA authorities in three health systems and putting forward recommendations that could be considered by French payers that could bring further innovations to French patients.

In scope here are all instruments which enable the measurement of health-related quality of life (including, therefore, a number of patient-reported outcomes). We exclude patient-reported experience measures insofar as they do not measure health-related quality of life but rather patient experience of the treatment, and utility values, again insofar as they do not measure health-related quality of life (this helps to explain why our analysis of NICE appraisals is not as in-depth as that undertaken for HAS and IQWiG, given NICE’s focus on utility values rather than HRQoL as more strictly defined here).

## Methodology

### Literature review

We conducted a literature analysis of relevant HAS, IQWiG and NICE documents published since 2018 across all therapeutic areas, including their official published methodological guidelines. Moreover, we reviewed recommendations and literature from key discussions around HRQoL evidence in the HAS appraisal process. See Additional file 1: Supplemental Methods for the full list of documents reviewed.

### Health technology assessment appraisal analysis

We undertook an analysis of the body within HAS which is responsible for the clinical assessment part of the appraisal, the Transparency Committee (CT; as compared with the economic assessment), and IQWiG appraisals dated 1 January 2019–31 December 2021 for drugs indicated for the treatment of rare diseases, respiratory diseases, oncology, cardiovascular diseases, metabolic, endocrinology and paediatric diseases, as accessible from PrismAccess (Prioritis proprietary database). We noted, for each appraisal whether HRQoL evidence was included in the dossier submitted, what instruments were used, whether the HRQoL evidence was considered sufficiently appropriate to be taken into account in the assessment and the reasons for rejecting this data where this was not the case. Our analysis therefore included all products reviewed by HTA, regardless of whether they were reviewed by both HAS and IQWiG or not (and did not conduct a direct comparative analysis of each product review by both agencies). Due to the wide difference in methodology in how NICE conducts its appraisal, and in the type of evidence requested by NICE (as it compares with HAS and IQWiG), we did not include NICE appraisals in this analysis.

### Expert opinion survey and interviews

We conducted a survey of 14 stakeholders, including patient advocates, healthcare providers, former HTA directors and methodologists (five from France, five from the United Kingdom and four from Germany). Topics covered by the survey included the principles that determine the types of HRQoL instruments accepted by HTAs, importance of HRQoL evidence for clinical versus economic appraisal and preferred instruments as well as an open question on how HRQoL evidence is assessed in the three countries. To deep-dive into the topics above, we also conducted the survey with six HTA stakeholders from patient advocacy groups and former HTA directors and three patient advocacy group (PAGs). See Additional file 1: Methods for an anonymized list of experts surveyed and interviews, as well as the questions asked in the survey.

### Advisory boards to consolidate opinions

We conducted two advisory boards with the leading experts co-authoring this report, with the aim of (1) understanding the benefit of including HRQoL evidence in HTA appraisals for patients; (2) coming to a consensus regarding the pros and cons of the current landscape in France, Germany and the United Kingdom; and (3) discussing consensus proposals to improve HRQoL evidence adoption France. Leading experts included HTA experts (three from France, one from Germany and one from the United Kingdom), clinicians (one from France), methodologists (three from France) and PAGs (two from France).

## Results

### The doctrine of the Commission de la Transparence (CT) emphasizes the importance of quality of life (HRQoL) data but does not include much detail

**Fig. 1:**
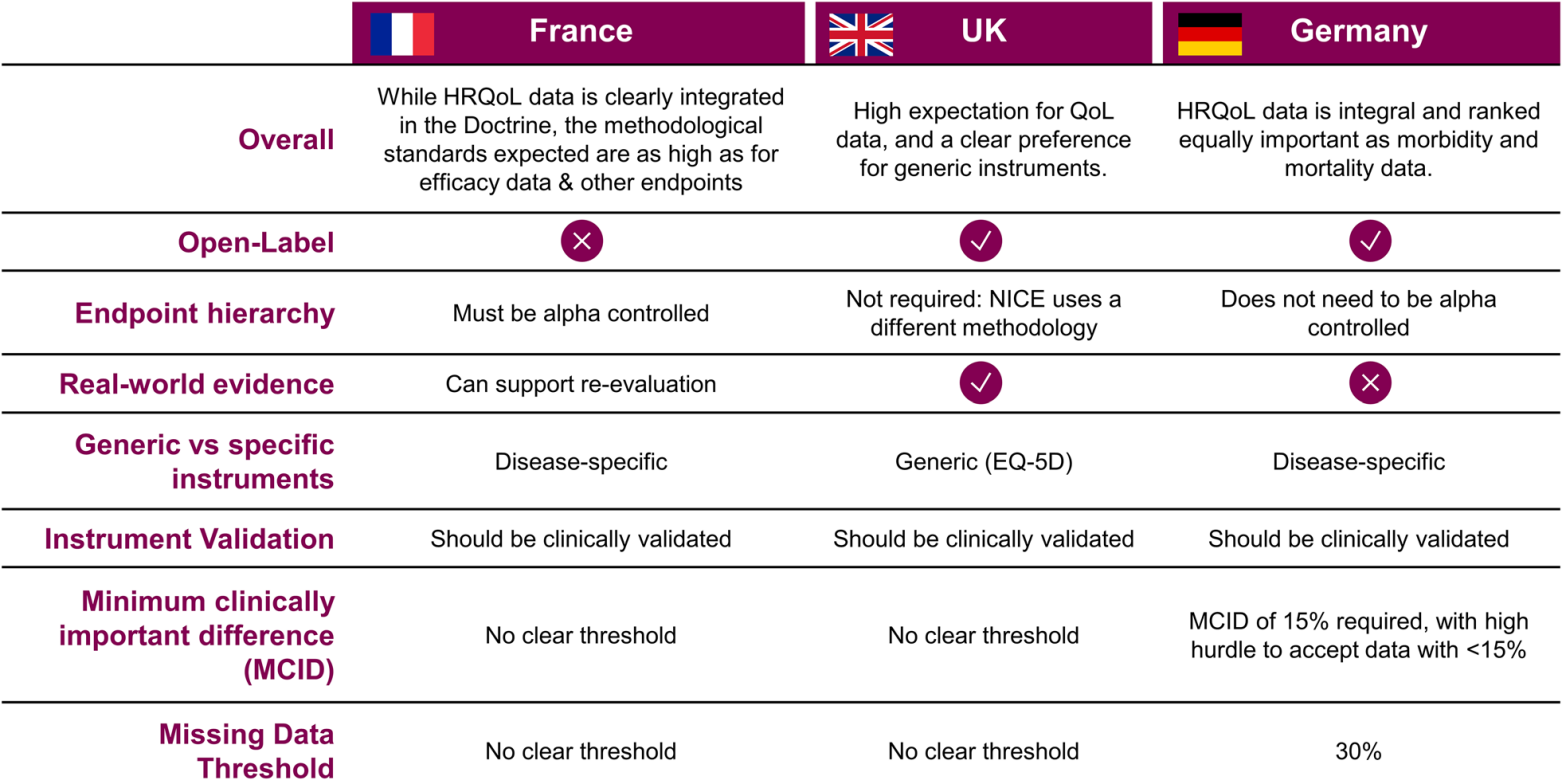
The three countries studied differ substantially in the HRQoL scales preferred and in how this evidence is assessed. Comparison of HAS [12], NICE [23] and IQWiG [15] methods guides, including an overall assessment of the approach, acceptance of open-label data, requirements for end-point hierarchy, acceptance of real-world evidence, preference of generic or disease-specific HRQoL scales, requirements for instrument validations, thresholds for minimum clinically important difference (MCID) and guidelines for missing data

Our analysis of methods used to assess HRQoL data in HTA appraisals across the United Kingdom, France and Germany showed how complex this assessment is (Fig. 1). This is complicated by the fact that no two therapeutic areas measure HRQoL are similar; indeed, even within oncology, substantial differences between cancer types mean that different HRQoL instruments will be used, and a uniform approach is difficult to put forward as a result. These analyses were used as a key input into the development of our proposals, which may help the adoption of HRQoL data. The HAS doctrine states “the quality of life data contributes to the evaluation of the clinical efficacy of a medication”. It also further states that “by completing the efficacy and safety data and dependent on medical context, quality of life can lead to an Amélioration du Service Medical Rendu (ASMR) superior to V in the case where they are: using validated and adapted instruments (preferably disease specific), and a rigorous methodology [12] [objective and minimum clinically important difference (MCID) specified in the protocol, double-blind, control of Type-1 error, appropriate frequency of measurement, appropriate time and study duration and extent of missing data”].

### Recent case studies show the positive impact which quality of life (HRQoL) data can have on the HTA appraisals

Recent case studies suggested by our ex-HTA directors advisors (Table 1) show the important impact which HRQoL data can have on HAS appraisals, across therapeutic areas (examples here include polyneuropathy, atopic dermatitis and protoporphyria). This often comes from HRQoL-specific end-points in randomized clinical trials (as in the examples of inotersen and dupilumab), but can also come from real-world evidence trials (as in the case of afamelanotide), though HTA attach different importance to real-world evidence (see Fig. 1 for a summary).

**Table 1:** Recent case studies show the impact which HRQoL evidence can have on a product’s benefit rating in France and Germany

Case study	HAS appraisal	NICE appraisal	IQWiG and G-BA appraisals
Treatment: DUPIXENT (dupilumab) Indication: atopic dermatitis Instrument: DLQI	Comparative study between treatment (*n* = 107) and placebo (*n* = 108) groups. Significant impact on the DLQI was demonstrated in the trial and was used as secondary judgement criteria by CT	NICE assessed similar data as that reviewed by HAS. The committee agreed that the product improved both efficacy and quality of life as measured by DLQI at week 16	IQWiG and G-BA also assessed the CHRONOS study (see HAS column). G-BA determined a considerable added benefit for dupilumab, based on significant improvements in morbidity (itch, sleep, eczema severity) and QoL (DLQI)
Treatment: TEGSEDI (inotersen) Indication: polyneuropathy Instrument: NORFOLK QoL-DN (questionnaire) and SF-36 (questionnaire)	Comparative study between treatment (*n* = 113) and placebo (*n* = 60) groups. Modest impact on the NORFOLK QoL-DN was demonstrated in the trial and was used as secondary judgement criteria by CT	NICE reviewed the data from the NEURO-TTR study. A statistically significant difference in favour of inotersen was seen on the mNIS + 7 but not on the Norfolk QoL-DN score The committee concluded that the evidence showed that inotersen had considerable benefit in slowing disease progression, but it did not stop progression	IQWiG and G-BA assessed the NEURO-TTR study and found a non-quantifiable added benefit for inotersen. This was driven by the functional domain of the SF-36. While the overall results on NORFOLK QoL-DN and SF36 were statistically significant, G-BA did not find the magnitude of the effect clinically relevant. No benefits were found on mortality and morbidity
Treatment: SCENESSE (afamelanotide) Indication: erythropoietic protoporphyria Instrument: DLQI and EPP-QOL	RWE data collection study following 117 patients over 2 years and evaluating the change in QoL over time**.** The EPP-QOL score had a mean QoL was significantly better in the treatment group. CT used these RWE findings	NICE considered the data emanating from the EPP-QoL questionnaire, as well as from the DLQI questionnaire, but noted some serious reservations about the collection method The committee concluded that the there was no marked improvement in the quality of life of patients who had treatment beyond the duration of the controlled clinical trials	IQWiG and G-BA found a non-quantifiable added benefit based on the “time spent in sunlight” as a morbidity end-point from the CUV039. G-BA reviewed the QoL data but concluded that the instruments were not validated in this disease and criticized that different versions of the instrument were used in the study

Case studies, selected by our ex-HTA directors, show the impact that HRQoL evidence can have on the benefit rating granted by HAS in France [44–47], NICE in the United Kingdom [48–50] and IQWiG and the federal joint commission (G-BA) [51–53] in Germany, across all therapeutic areas, and using a wide array of HRQoL scales. Abbreviations: DLQI, Dermatology Life Quality Index; G-BA, German federal joint committee; IQWiG, Institute for Quality and Efficiency in Health Care; QoL-DN, Quality of Life – Diabetic Neuropathy; RWE, real-world evidence; SF-36, 36-item Short Form Survey

Overall, these examples demonstrate the importance of including HRQoL data in the value assessment, and how highly HTA value this data when it is collected according to guidance.

### A systematic review of recent HAS and IQWiG appraisals shows that the HAS’s methodology leads to a higher rate of rejection of HRQoL data

**Fig. 2:**
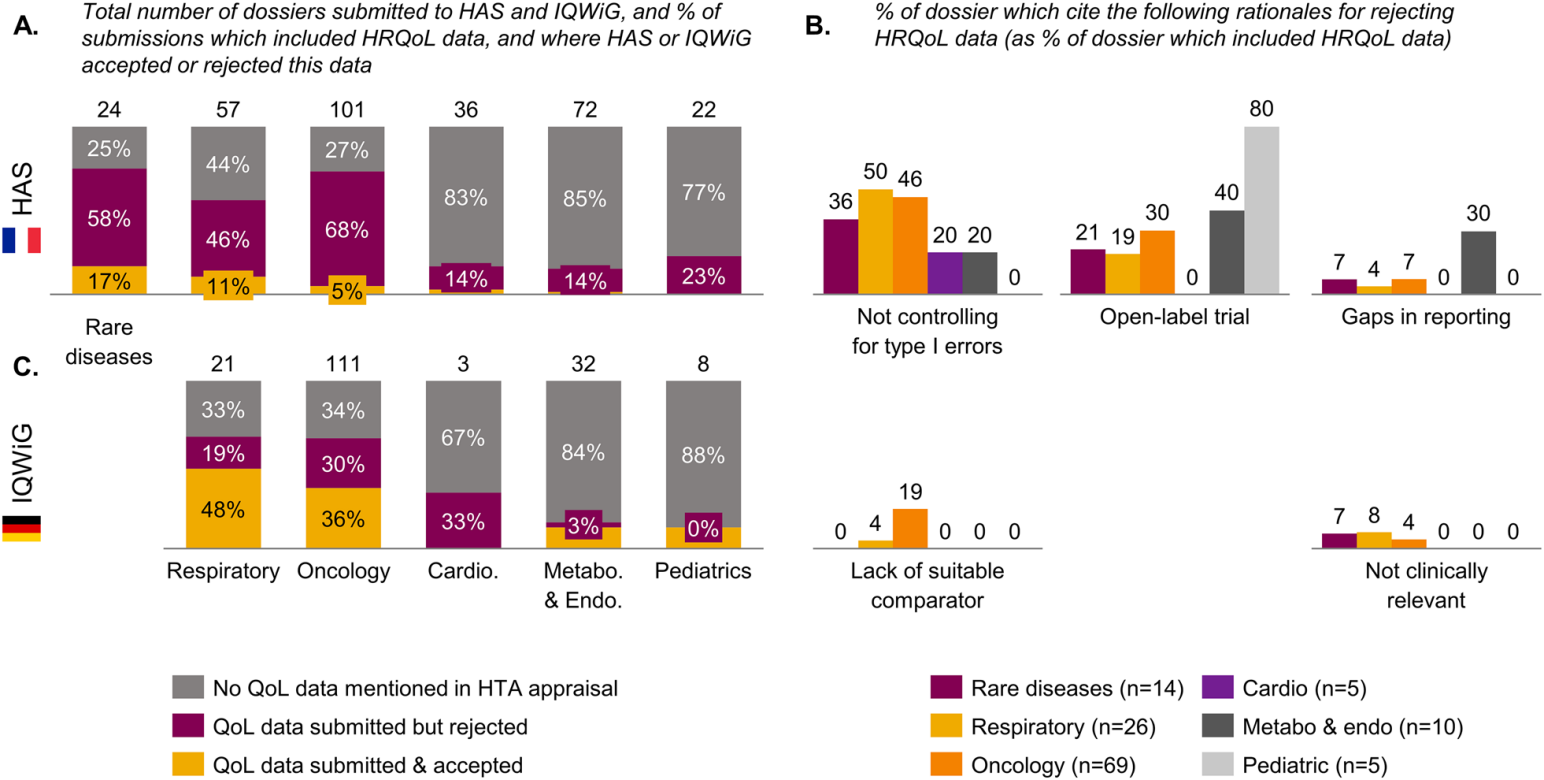
Analysing recent HTA appraisals shows HAS’s methodology to lead to a higher rate of rejection HRQoL data than IQWiG. **A** Number submissions to HAS across disease areas which included HRQoL data, and when this was accepted. **B** Rationale quoted by HAS for rejecting HRQoL data as a percent of submissions including HRQoL data across disease areas. **C** Number of all submissions to IQWiG across disease areas which included HRQoL data, and when this was accepted. HAS and IQWiG appraisals between 1 January 2019 and 31 December 2021 were analysed across six therapeutic areas (rare disease, respiratory disease, oncology, cardiovascular diseases, *metabolism/endocrinology and paediatrics). Appraisals were collected from PrismAccess. Reasons for rejection are not mutually exclusive; some appraisals mentioned more than one reason for rejecting HRQoL evidence. **These reasons for rejection commonly cause the data to be regarded as “exploratory”, which is the most frequent cause of HRQoL data rejection mentioned in HAS appraisals. IQWiG does not undertake an assessment for rare diseases; this is done by the German federal joint committee (G-BA). NICE’s methodology is different, as it usually undertakes the clinical and economic assessment jointly, hence NICE appraisals are not reviewed here

We logged every HAS opinion from 2019, 2020 and 2021 and assessed the share of dossier which included HRQoL data as well as the HAS consideration of this data (due to the wide difference in methodology in how NICE conducts its appraisal, and in the type of evidence requested by NICE as it compares with HAS and IQWiG, we did not include NICE appraisals in this analysis). We undertook this analysis for six therapeutic areas rare diseases, paediatrics, oncology, metabolism and endocrinology, cardiovascular diseases, and respiratory diseases assessed between 1 Jan 2019 and 31 Dec 2021. The share of dossiers which included HRQoL data varied widely by therapeutic area (TA), from 16% to 75% (Fig. 2A). HRQoL data was more frequently collected and reviewed by HAS in rare and respiratory diseases. Cardiovascular, metabolism and endocrinology as well as paediatric clinical appraisals had far fewer submissions with HRQoL data submitted, potentially due to the non-chronic nature of disease or challenges with caregiver HRQoL data collection. Across disease areas, HRQoL data was submitted and accepted by HAS in < 20% of all appraisals. Specifically, the proportion of assessments that considered HRQoL data was 17% in rare diseases (*n* = 24), 11% in respiratory (*n* = 57), 5% in oncology (*n* = 101), ~3% in cardiovascular (*n* = 36), ~1% in metabolism and endocrinology (*n* = 72) and 0% in paediatrics (*n* = 22).

Looking at the HAS opinions in more detail, the most frequent reasons for rejecting the data were methodological, usually because the analysis was considered exploratory, as it was not included in the statistical testing hierarchy (Fig. 2B). The common reasons for rejection did not vary substantially by therapeutic area: lack of control for alpha risk is the most common cause (closely linked to statistical testing hierarchy). The second most common cause for rejection was the open-label nature of trials; other reasons include gaps in reporting, lack of suitable comparators and not clinically relevant or missing statistical tests.

Indeed, the HAS doctrine states that HRQoL data collecting must be done rigorously, according to the following criteria: “using validated and adapted instruments (preferably disease-specific), and a rigorous methodology (objective and MCID specified in the protocol, double-blind, control of alpha risk, frequency, appropriate time and duration and limited missing data” [12].

To provide an international benchmark, we looked at IQWiG’s appraisal over the same time-period, in the same therapeutic areas (Fig. 2C). The percentage of appraisals that included HRQoL data did not vary substantially compared with dossiers submitted to HAS. The most striking difference observed was in the share of a dossier where the HRQoL data was reviewed and accepted as valuable by IQWiG: up to 48% of dossiers for respiratory diseases, and 36% in oncology (versus 11% and 5% at HAS for the same therapeutic areas). The main reasons for rejecting data were gaps in reporting (60% of rejections in oncology), with relevance of HRQoL scales used as the second most frequent reason (18% in oncology). The main difference between the IQWiG and HAS’s approaches seemed to be IQWiG’s acceptance of data outside of the testing hierarchy, and the fact that IQWiG was more open to data from open-label trials or real-world evidence studies. Both IQWiG and HAS reject data where there are too many reporting gaps; IQWiG specifies a threshold of 70% of data required for the statistical analysis, while HAS does not specify a threshold.

**Fig. 3:**
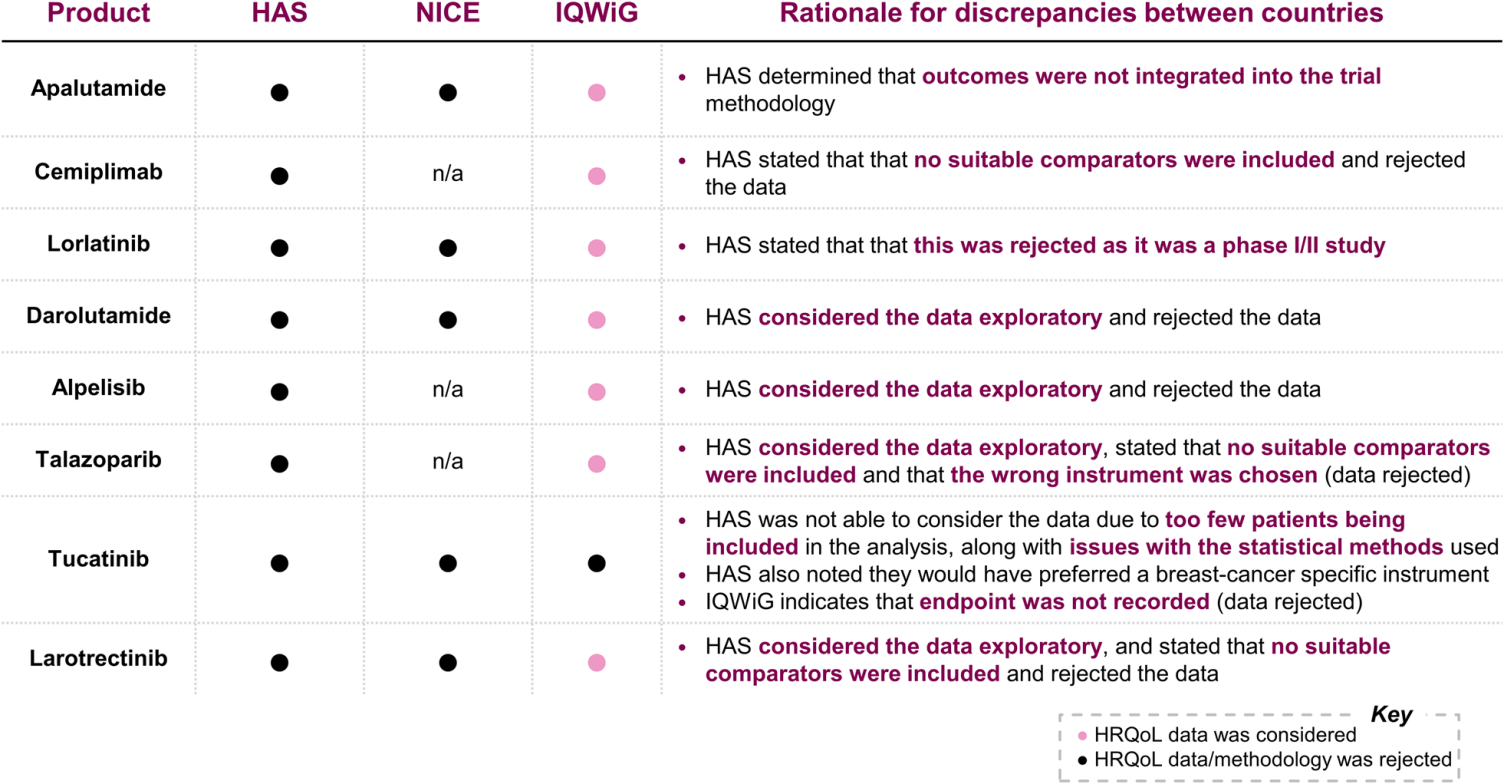
Recent case studies show the difference in how HAS and IQWiG handle HRQoL evidence in dossiers submitted to them. Case studies, selected by our ex-HTA directors, show, for HAS [24–31], NICE [32–35] and IQWiG [36–43], whether the HRQoL data submitted was considered or not, and the rationale for the discrepancies. Note: NICE did not review all the products that HAS and IQWiG reviewed, this is indicated by “n/a”

This is illustrated by examples such as apalutamide, cemiplimab and lorlatinib, where HAS considered the statistical analyses exploratory, while IQWiG decided to consider the statistical analyses in the appraisal (Fig. 3). Nonetheless, HAS is generally stricter than IQWiG in its consideration of HRQoL evidence, as shown by its frequent rejection of trials without “suitable comparators” and frequent assessment that data were exploratory. NICE merges the economic and clinical appraisal into a single assessment, while HAS and IQWiG separate the two analyses, leading to different methodologies for assessing HRQoL.

### Survey results show that HRQoL evidence from a validated instrument is seen as essential, but fit with guidance is the greatest barrier to wider adoption in France

**Fig. 4:**
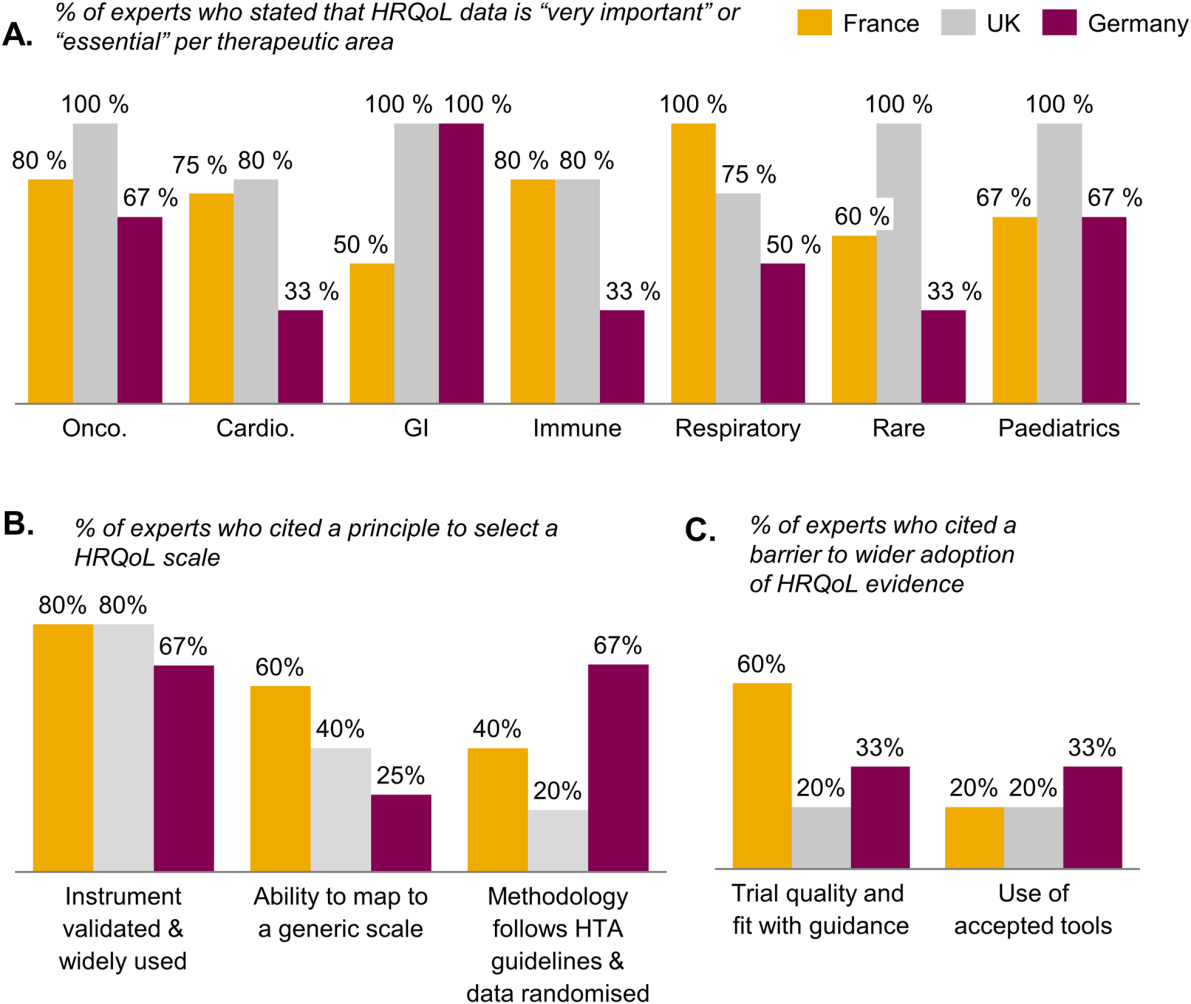
Survey results show that HRQoL evidence from a validated instrument is seen as essential but fit with guidance is the greatest barrier to wider adoption in France. Data from a survey of 14 ex-HTA directors (5 ex-HAS in France, 5 ex-NICE in the United Kingdom and 4 ex-IQWiG in Germany) between the 21 and 31st of January. See Additional file 1: Methods for the detailed list of questions asked. **A** Percent of experts who stated that HRQoL data was “essential” or “very important” for each of the therapeutic areas listed; **B** percent of experts who stated that the following principles determined the acceptability of a HRQoL instrument; **C** percent of experts who stated the following as the most important barriers to further uptake of HRQoL data in their respective countries

According to our survey, a majority of experts believed that HRQoL data is an essential part of the clinical assessment undertaken by HTA in France, the United Kingdom and Germany (Fig. 4A), though there was some variation between countries and therapeutic areas (for example, German ex-HTA directors were less likely to see HRQoL data as essential, compared with French ones The use of a validated and widely used HRQoL instrument was the main principle which determined HRQoL data acceptability across the three countries surveyed, though in Germany the importance of following HTA guidelines closely was also an important factor (indeed, IQWiG’s methodological guidelines are much more detailed than those issued by, for example, HAS [12, 15]; Fig. 4B). When asked about the main barriers to further adoption of HRQoL data, experts stated that trial quality and fit with the methodological guidance issue was the main barrier in France (thereby echoing the German ex-HTA directors; Fig. 4C).

To discuss potential opportunities to improve HRQoL data adoption, we setup an advisory board, who framed four areas that determine HRQoL data acceptability: (1) patient perception, (2) end-point hierarchy, (3) trial design, and (4) data collection.

(1) Importance of patient perception: Delivering patient-centric medicines will require including the patient voice and patient reported data in the value assessment. PAGs clearly stated, both through our interviews and AdBoards, that taking the patient’s quality of life into account in the value assessment is critical to making sure their voice is heard and the patient impact is understood. Indeed, PAGs strongly believe these should be considered in reimbursement decisions. A majority of French ex-HTA directors surveyed believe that HRQoL is essential to the clinical process (100% for immune disease, 80% for gastrointestinal diseases, 75% for oncology, 67% for paediatric diseases, 60% for respiratory diseases and 50% cardiovascular diseases). However, in AdBoard discussions, it became clear that there was a perception that HAS ranks HRQoL secondary to safety and efficacy in its value assessment. Additionally, the minimum meaningful difference is an important threshold to define and reach as part of the value assessment process; indeed, there was a sense from our AdBoards that this can be an abstract number which differs by instrument and can be difficult to assess as part of the clinical appraisal, or even by expert methodologists. Patients may be best placed to contextualize the HRQoL data collected and help input on the minimal meaningful difference observed. In Germany, patient advocacy groups provide insight into the relevance of data to patients going into the clinical appraisal process.

(2) Testing hierarchy: The exploratory nature of most HRQoL data submitted in the clinical appraisal process is currently the most frequent reason for this data to be excluded. Indeed, in our survey, methodology and instrument validation were cited by > 80% of French HTA experts as one of the key principals behind HRQoL data acceptance in the clinical appraisal. One such methodological requirement is to account for Type-1 error (multiplicity of analyses) by including HRQoL end-points in the hierarchy of end-points and in the pre-specified statistical analysis plan. Experts at our AdBoards highlighted that, to demonstrate benefit on efficacy, safety or HRQoL, requires data to meet the same requirements. Input from IQWiG methodological guidelines and German experts in interviews showed that the approach there was different: while both bodies will only review secondary data pre-specified in the statistical analysis plan, they do not mandate inclusion in the testing hierarchy.

(3) Trial design: HRQoL data from open-label modalities in quantitative trials are not currently considered by HAS, likely due to their more subjective nature. The HAS doctrine clearly states that HRQoL data must be from a double-blind trial to be accepted in the appraisal. However, sometimes double-blind trials are not possible due to ethical constraints. Additionally, our survey results showed that 90% of HTA experts agree that randomized clinical trial data is superior to, and more important than RWE data, experts agreed that RWE HRQoL data is a useful addition to existing trial designs and should be considered by HAS (particularly in the case of accelerated/managed entry agreements). In the Germany, according to experts, RWE is accepted for HRQoL data as part of the clinical appraisal process: the IQWiG doctrine also states that “There is a preference for trials which ‘combine proximity to everyday conditions… such as real world trials, practical trials or pragmatic trials’ however there must be a high certainty of results” [15].

(4) Data collection: High-quality data collection is key, but missing data and choosing the right instrument is often a challenge. Without high-quality data, statistical analysis and comparisons in the appraisal process are not meaningful. Across the United Kingdom/France/Germany data collection must be undertaken with well-validated, relevant instruments and as little missing data as possible (Germany usually uses a 30% threshold for missing data/70% threshold for complete data). Selecting the right instrument, which is relevant to the patient, easy to complete, and defines the appropriately validated aggregate score or sub-score to analyses in the pre-fine statistical analysis plan are all key to enabling HAS to evaluate the HRQoL data (the point in time when the data is collected is often a key question). This is particularly salient as many of the instruments are decades old, and not relevant to patients today, or to the targeted therapies increasingly used in clinical trials. During our AdBoards, PAGs explained that the length of some HRQoL questionnaire create real barriers to patients completing these forms and having their preference considered.

## Discussion

### Based on these four areas of work, our advisory board put forward a list of 10 proposals each to industry and HAS to drive HRQoL data adoption.

We discussed a number of proposals, which are detailed below (Table 2). These proposals are very much intended as collaborative propositions to HAS, as well as actions we believe the industry could and should take on to improve adoption of HRQoL data in France. Our proposals are segmented among both short- and long-term horizons. Though we realize some of our proposals may involve complex long-term discussions, we strongly believe these would highly benefit patients, as well as create a positive feedback loop between HAS and industry. See Additional file 2: Table S1 for more detail on our proposals.

**Table 2:** Table of 10 proposals to industry and 10 proposals to HAS to improve HRQoL data adoption in France, framed around the four key areas of work: patient perception, testing hierarchy, trial design and data collection

Proposals to industry	Proposals to HAS
Patient perception	
Proposal 1.1: Establish the minimal clinically important difference or meaningful change for each relevant patient population	Proposal 2.1: Improve the input PAGs are able to have in the assessment process by asking them to comment on industry submissions (including on the difference observed in clinical trials), possibly by providing detailed guidelines on patient engagement allowed in the current legal context
Proposal 1.2: Seek further input from patient advocacy groups when designing trials (specifically in selection of outcomes measures)	
Proposal 1.3: Where legislation allows, share relevant literature and data with patient advocacy groups	
Testing hierarchy	
Proposal 1.4: Remind clinical trial teams of the importance of collecting HRQoL data	Proposal 2.2: Provide more feedback on the quality and appropriateness of HRQoL data submitted, and any challenges to be addressed in future trials
	Proposal 2.3: Where possible, consider HRQoL data that was not included in a testing hierarchy
Proposal 1.5: Clarify the HRQoL score(s) of interest upfront in the stats analysis plan	Proposal 2.4: Consider the value of HRQoL data from RWE studies (and, if relevant, develop appropriate guidance on how to obtain, analyse and report RWE data, and clarify the circumstances in which these would be most useful)
Trial design	
Proposal 1.6: Minimize the risk of a bias when collecting HRQoL data in open-label trials	Proposal 2.5: Define specific circumstances where using HRQoL data from open-label trials would be acceptable
Proposal 1.7: Submit detailed rationales for using HRQoL data from open-label trials	Proposal 2.6: Clarify the guidance for the type of instrument to use
	Proposal 2.7: Consider the role of novel trial designs and their role in collecting HRQoL data in future studies
Data collection	
Proposal 1.8: Use digital technologies where this could improve the quality of HRQoL data or the patient experience	Proposal 2.8: Clarify the relative importance of HRQoL versus other clinical parameters such as efficacy
Proposal 1.9: Simplify HRQoL instruments used by reducing their length, and improve relevance to patients	Proposal 2.9: Consider differential recommendations for products where they have a similar efficacy but different impact on patient HRQoL
Proposal 1.10: Thoughtfully choose appropriately validated instruments used in trials, and clearly report on these	Proposal 2.10: Support the definition of a Europe-wide threshold for missing data as part of EUnetHTA, or discuss methods that will enable the decrease of the impact of missing data in industry submissions

In this study we analysed the methodological guidelines published by HTAs and NICE, as well as the appraisals published across six therapeutic areas in 2019–2021 for HAS and IQWiG. HAS and IQWiG are closer in terms of process, separating the clinical and economic assessments as opposed to NICE; though as we showed there are still important differences in their methodology [12, 15]. While NICE, HAS and IQWiG recommend using validated, disease-specific instruments, our analysis of recent appraisals shows IQWiG to be more likely to review HRQoL data submitted than HAS, across therapeutic areas. The methodological guides published by the HTA outline the differences in standards for data accepted: for HAS’s CT, HRQoL end-points must be included in the testing hierarchy, while IQWiG will at times accept data from end-points outside of the hierarchy. In addition, while IQWiG puts HRQoL data on the same level of importance in its assessment as mortality and morbidity, which therefore opens the door for a broader perspective than pure clinical efficacy [15], the HAS CT doctrine simply states that rigorous demonstration of quality of life benefits may increase the “ASMR” (clinical added value) given to a product, thereby limiting the scope of the assessment to pure clinical efficacy [12].

Little has been published about the state of HRQoL data as part of the HTA assessment in France, though recently recommendations were published from the Ateliers de Giens, which bring HTA, industry and methodologists together in France [16]. Roussel et al. concur that a lot of work has been undertaken recently by HAS to understand the current field (for example, the recent publication on international best practise [8]), and have put forward three recommendations: “(1) Better information for all parties involved in a dossier for technology assessment, (2) Systematization of the collection of PROMs for evaluation of health products, (3) Improved quality of dossiers thanks to the use of relevant and validated tools”, which go in the same directions as our proposals 1.2, 1.5, 1.8, 1.9, 2.1 and 2.2.

There are a number of recent studies debating the possibility of a bias in PROs collected as part of open-label trials (see proposals 1.4, 1.5, 2.4). While most retrospective analyses find no bias in favour of the experimental arm in PRO data collected as part of open-label trials [17–20], one study from 2019 found evidence of some biases particularly where there were large differences in completion rates between the control and experimental arms [21]. Reviewing PRO data from open-label data would be important where randomized trials are not feasible, but it will be important to minimize this risk (proposal 1.4) and discuss with HAS about specific circumstances where open-label data would be most impactful (proposal 2.4). Further investigations of the method used by IQWiG may also be helpful here, given their experience in accepting open-label data for some time now (Figs. 3 and 4).

We also propose investigating the use of synthetic arms (for example, in specific circumstances where appropriate controls may not be feasible), as recent publications suggest such trial types may be impactful if designed correctly and with appropriate caveats [22].

Our 20 proposals to both industry and HAS are an attempt at creating a positive feedback loop between the two stakeholders and the wider community (healthcare providers and patient advocates in particular). Some industry players are already putting some of the proposals put forward here in place, in particular to seek broader input from patient representatives in clinical trial designs including end-points and to collect HRQoL data more comprehensively as part of their clinical trials (proposals 1.2, 1.8 and 1.9). To go further, we encourage industry to work with PAGs to identify any particular therapeutic areas for which measurements are most important and improve and validate the existing instruments to make them more relevant to patients (proposals 1.2 and 1.7).

We also defined proposals for HAS. As with the industry, HAS is already addressing some of these (for example, proposals 2.1, 2.6 and 2.10) but we would encourage further acceleration to better appreciate of the value of HRQoL data. We recognize that some areas will be easier to address than others for HAS: some proposals are short term (proposals 2.1 to 2.2), while others are more long-term areas of work which will require engagement with the wider community and discussions to mature (2.6 to 2.10).

To implement our proposals, we highlight the importance of the wider community (for example, patient advocacy groups, healthcare providers, Ministry of Health), without whom none of our proposals could be implemented. Nonetheless, enabling broader utilization of HRQoL data could have benefits in the value assessment of medicines. Our work only looked at six therapeutic areas (rare diseases, respiratory diseases, oncology, cardiovascular diseases, metabolic/endocrinology and paediatric diseases) and focused on a narrow set of 3 years’ worth of appraisals published by two HTA in Europe (HAS and IQWiG). We focused our study on the clinical assessment, to the exclusion of the economic appraisal undertaken by both HAS and IQWiG (or the joint assessment undertaken by NICE).

## Conclusions

We believe that collaborating on implementing these actions and proposals will enable the quality of life of patients to be more widely considered in the appraisal process, especially in therapeutic areas where efficacy is not the only factor which impacts patient well-being. We also believe that mobilizing the wider group of stakeholders will be critical to implementing our proposals and driving up adoption of HRQoL data. As a next step, both the industry and HAS should seek to encourage discussion and detailing of these proposals in relevant meetings and publications with these stakeholders so that they can be effectively implemented within the French system.

### Supplementary Information

**Additional file 1:** Please find additional, more detailed methods in our Supplemental methods file.

**Additional file 2:** Please find additional detail on our 20 proposals in the additional data.

## Data Availability

Most of the data used in this study is publicly available on the HAS, IQWiG and NICE websites. Survey data are available from the corresponding author upon reasonable requests.
