# Supplementary material for: Opportunities to improve the adoption of health-related quality of life evidence as part of the French Health Technology Assessment process
Source: Health Res Policy Syst. 2023 Dec 19;21:137. doi: 10.1186/s12961-023-01081-8 (PMC10729510; doi:10.1186/s12961-023-01081-8)
Supplement: Supplementary file 1 — Additional file 1: Please find additional, more detailed methods in our Supplemental methods file. [file 12961_2023_1081_MOESM1_ESM.docx]

**Supplemental Methods**

***List of methodological guidelines & other documents reviewed in this project***

| Document | Date | URL |
| --- | --- | --- |
| HAS guidance on PROMs | 2020 & 2021 | [URL](https://www.em-consulte.com/article/1502438/article/place-des-mesures-rapportees-par-les-patients-prom)  [URL](https://www.has-sante.fr/upload/docs/application/pdf/2020-05/panorama-proms-prems_note_de_cadrage.pdf) |
| Ministry of health letter to HAS | October 2021 | [URL](http://www.nile-consulting.eu/drop/21104-Lettre_de_saisine_OV-HAS.pdf) |
| HAS: guidance on economic assessment | July 2020 | [URL](https://www.has-sante.fr/upload/docs/application/pdf/2021-09/doctrine_de_la_ceesp.pdf) |
| HAS survey on PROs | December 2021 | [URL](https://www.has-sante.fr/upload/docs/application/pdf/2021-12/maquette_questionnaire_has_enquete_proms_revu_sc.pdf) |
| IQWiG General Methods 6.0 | November 2020 | [URL](https://www.iqwig.de/methoden/general-methods_version-6-0.pdf) |
| Indicators of the quality of healthcare provided | April 2022 | [URL](https://www.has-sante.fr/jcms/p_3306659/fr/indicateurs-de-qualite-des-parcours-de-soins) |
| 10-year strategy for the fight against cancer (2021-30); guidance for 2021-25 INCa; |  | [URL](https://solidarites-sante.gouv.fr/IMG/pdf/feuille_de_route_-_strategie_decennale_de_lutte_contre_les_cancers.pdf) |
| HAS guidance for RWE studies | June 2021 | [URL](https://www.has-sante.fr/upload/docs/application/pdf/2021-06/real-world_studies_for_the_assessment_of_medicinal_products_and_medical_devices.pdf) |
| HAS guidance on assessing products for breakthrough designation | April 2022 | [URL](https://www.has-sante.fr/upload/docs/application/pdf/2021-06/acces_precoces_-_doctrine.pdf) |
| CHTE Methods Review, Health-related quality of life | July 2020 | [URL](https://rees-france.com/wp-content/uploads/2020/12/2020-CHTE-2020-Health-related-quality-of-life-.pdf) |
| HAS guidance on product assessment | December 2020 | [URL](https://www.has-sante.fr/upload/docs/application/pdf/2021-03/doctrine_ct.pdf) |

***Anonymized list of stakeholders who participated in our project***

Stakeholders who participated to our survey:

- France:
  - Previously a Board Member & Consultant for HAS (CT) for 7 years
  - Previously a was member of HAS (CT) for 6 years
  - Previously Head of the Drug Evaluation Service at HAS for 12 years.
  - Previously a pharmacist who was an HTA Project manager at HAS for 1 year. This member was also previously at NICE
  - Previously a member of HAS (CT) for 5 years
  - Previously a member of HAS (CT) for 9 years
- Germany:
  - Previously at DIMDI for 12 years until recently in the position of Head German Agency for HTA
  - Previously worked with GKV and G-BA as a Policy Consultant on Pricing and Reimbursement
  - Previously a reimbursement specialist and advisor at G-BA for the past 20 years*
  - Previously a policy advisor and member of GKV-Spitzenverband (16 years)
- United Kingdom:
  - Previously a director at NICE (5 years)
  - Previously a member of the NICE Technology Appraisal Committee (6 years)
  - Previously a Health Policy Advisor at NICE (9 years)
  - Previously was Head of Scientific Advice at NICE (4 years)
  - Previously a committee member at NICE (1 year) and serves as an editorial board member at Pharmacy in Practice
  - Previously was a Medicines and Prescribing Associate at NICE (4 years)

Stakeholders who participated to our advisory boards:

- Germany: Ex - Head of Department for Methods of Community Medicine at a Germany university
- United Kingdom: Professor of Health Economics in the School of Health and Related Research at a university in the United Kingdom
- France:
  - Former Head of Drug Evaluation Department at HAS
  - Former director of Scientific Affairs at LEEM
  - Professor in Biostatistics at a French university and previously a member of a HAS committee
  - Emeritus professor at a French Business School and Scientific advisor to the Ministry of Health
- Statistician at a French university, specializing in cancer patient HRQoL
- Professor of Therapeutics, Health Economics Clinical Trial Unit at a French hospital
- Vice-president of a large French patient advocate group
- Medical director of a large French hospital
- Manager at a French patient advocate group

***Detailed survey questions***

1A. What is your name?

1B. Which of the following bodies do you have experience with (e.g., a prior position of >2 years)? *Please select multiple if applicable*

1C. In which countries do you have experience?

1D. With which Therapeutic Areas are you familiar? Please tick your level of expertise below.

2A. Are there existing guidelines for HRQoL Data (e.g., PROM/PRO, PREM or other) in your country's HTA body? If so, please provide a link if possible.

2B. How often is HRQoL data (e.g., PROM/PRO, PREM or other) in the submission file taken into account in the HTA assessment in your country?

2C. From your knowledge, what is the relative importance of HRQoL data compared to safety data and efficacy data?

2D. What are the benefits of including HRQoL data in your HTA submission?

2E. If HRQoL data was exploratory in clinical trial, how is it taken into account if considered by your HTA body?

2F. Is your HTA body placing more importance on clinical trial HRQoL data or Real-world evidence (RWE) HRQoL data?

2G. If RWE HRQoL data is taken into account, which methodologies are used? How does its importance compare to clinical trial data?

2H. How important are Quality of Life instruments generally for **clinical assessment** of the following Therapeutic Areas (TAs)?

2I. How would you value HRQoL data for rare diseases and for Pediatric populations? Please explain your response.

2J. To what extent are generic HRQoL instruments more or less relevant than disease-specific ones for **clinical assessment** of the following Therapeutic Areas (TAs)? Please explain your response below.

2K. For which TAs/disease groups is HRQoL data not relevant? Why?

2L. What are examples of caregiver HRQoL data being submitted or considered as part of the HTA assessment?  For any examples relating to pediatric populations, how does the importance of the caregiver HRQoL change?

2M. What are the principles that determine which HRQoL instruments can be used as part of the clinical assessment?

2N. Which specific instruments are preferred by the HTA body with which you are most familiar?

2O. [Generic Instruments] What is the relevance (i.e., which instruments best demonstrate clinical impact of HRQoL improvement to HTA’s) of the following HRQoL instruments for the clinical assessment?

2P. If you selected "Other", please specify which HRQoL instruments you have identified as relevant.

2Q. For the Generic HRQoL instruments you have listed as (3) very relevant or (4) essential, what do you think are the **main strengths** as it relates to the **clinical assessment**?

2R. For the Generic HRQoL instruments you have listed as (3) very relevant or (4) essential, what do you think are the **main weaknesses**as it relates to the **clinical assessment**?

2S. For the Generic HRQoL instruments you have listed as (3) very relevant or (4) essential, what do you think are the **main barriers to industry submitting these instruments**? How could these be addressed?

2T. For the Generic HRQoL instruments you have listed as (3) very relevant or (4) essential, what do you think are the **main barriers to HTA reviewing data for these instruments**? How could these be addressed?

2U. [Oncology-Specific] What is the relevance (i.e., which instruments best demonstrate clinical impact of HRQoL improvement to HTA’s) of the following HRQoL instruments for the clinical assessment?

2V. If you selected "Other", please specify which HRQoL instruments you have identified as relevant.

2W. For the Oncology-specific HRQoL instruments you have listed as (3) very relevant or (4) essential, what do you think are the **main strengths** as it relates to the **clinical assessment**?

2X. For the Oncology-specific HRQoL instruments you have listed as (3) very relevant or (4) essential, what do you think are the **main weaknesses** as it relates to the **clinical assessment**?

2Y. For the Oncology-specific HRQoL instruments you have listed as (3) very relevant or (4) essential, what do you think are the **main barriers to industry submitting these instruments**? How could these be addressed?

2Z. For the Oncology-specific HRQoL instruments you have listed as (3) very relevant or (4) essential, what do you think are the **main barriers to HTA reviewing data for these instruments**? How could these be addressed?

2AA. [Illustrative Other TA HRQoL Instruments] What is the relevance (i.e., which instruments best demonstrate clinical impact of HRQoL improvement to HTA’s) of the following HRQoL instruments for the clinical assessment?

2BB. If you selected "Other", please specify which HRQoL instruments you have identified as relevant.

2CC. For the Illustrative other TA HRQoL instruments you have listed as (3) very relevant or (4) essential, what do you think are the **main strengths** as it relates to the **clinical assessment**?

2DD. For the Illustrative other TA HRQoL instruments you have listed as (3) very relevant or (4) essential, what do you think are the **main weaknesses**as it relates to the **clinical assessment**?

2EE. For the Illustrative other TA HRQoL instruments you have listed as (3) very relevant or (4) essential, what do you think are the **main barriers to industry submitting these instruments**? How could these be addressed?

2FF. For the Illustrative Other TA HRQoL instruments you have listed as (3) very relevant or (4) essential, what do you think are the **main barriers to HTA reviewing data for these instruments**? How could these be addressed?

2GG. What are novel HRQoL instruments or data collection technologies which you think will become important? What are the pros and cons of this/these metrics? Over what period will these instruments become important (1-3 years, 3-5 years ,5-10 years, 10+ years)?

2HH. Please assess the relative importance for addressing clinical need vs. quality of life for the following Therapeutic Areas (TAs).

3A. How important are Quality of Life instruments generally for **economic assessment** of the following Therapeutic Areas (TAs)?

3B. How does the choice of instrument differ when it comes to the economic assessment?

3C. Would an increased focus on the economic assessment impact the importance of HRQoL data?

3D. How could we use other patient-relevant aspects in the economic appraisal, e.g., patient preference?

4A. How do you think industry can help address barriers to adoption of HRQoL data as part of HTA assessment?

4B. What role do patients play in decision-making outside of providing patient-relevant outcome (PRO) data?

4C. Please provide best practice example(s) in industry’s collection, analysis, and interpretation of data prior to the HTA assessment. Please specify the below:

4D. Please provide best practice example(s) where HRQoL data positively impacted the clinical assessments. Please specify the below:

4E. Are you familiar with appraisals performed by the Haute Authorité de Santé (HAS), also known as the French National Authority for Health?

4F. How do you think we can best engage HAS & produce an impactful report?

4G. How do you expect the Transparency Committee's (CT's) position on HRQoL data to evolve in coming years?

4H. What are the main barriers to industry submitting HRQoL data to HAS?

4I. What are the main barriers to acceptance of HRQoL data for HAS?

4J. What might be some of the risks that HAS would need to mitigate?

4K. What role could pharma play in making adoption of HRQoL in France easier?

4L. How do you think the weight attached to HRQoL data in Amélioration du service médical rendu, or improvement to actual benefit (ASMR) assessment will change in coming years?

4M. What would be the top 3 levers to apply based on best practice in other markets for HAS to incorporate HRQoL in ASMR assessment?
