# Supplementary material for: Opportunities to improve the adoption of health-related quality of life evidence as part of the French Health Technology Assessment process
Source: Health Res Policy Syst. 2023 Dec 19;21:137. doi: 10.1186/s12961-023-01081-8 (PMC10729510; doi:10.1186/s12961-023-01081-8)
Supplement: Supplementary file 2 — Additional file 2: Please find additional detail on our 20 proposals in the additional data. [file 12961_2023_1081_MOESM2_ESM.docx]

**Supplemental Data**

| **Proposals to industry** | **Proposals to HAS** |
| --- | --- |
| **Patient perception** | |
| **Proposal 1.1: Establish the minimum clinically important difference or meaningful change for each relevant patient population**: When instruments are selected and positioned in the statistical analysis plan, industry should seek to establish a suitable minimum meaningful difference in collaboration with HAS and PAGs | **Proposal 2.1: Improve the input PAGs are able to have in the assessment process by asking them to comment on industry submissions (inc. on the difference observed in clinical trials), possibly by providing detailed guidelines on patient engagement allowed in the current legal context:** We propose to explore funding options to ensure PAGs can deliver this crucial role. To truly put the patient at the center of the assessment, we also propose to involve patients/PAGs more heavily in the assessment – as well as clarifying the role they play in this. For example, they could be asked to contextualize the HRQoL data presented and present the patient perspective on the data observed during the clinical trial (this could include interviewing patients and including their insights into the report presented). Another potential role could include seeking their input on the minimum meaningful difference required. Finally, providing PAGs with feedback on the input they provide could both (1) create a virtuous cycle whereby the quality of their input continuously improves and (2) motivate PAGs by showing them the impact their perspective has on product assessments |
| **Proposal 1.2: Seek further input from patient advocacy groups when designing trials (specifically in selection of outcomes measures):** During clinical trial design industry should seek to get input from PAGs and patients on endpoints / instruments to use to ensure they are patient-relevant (as well as one the minimum meaningful difference- see above) |  |
| **Proposal 1.3: Where legislation allows, share relevant literature and data with patient advocacy groups**: To enable better representation of the benefits of HRQoL data for patients by PAGs, industry should, where possible, share relevant studies and articles |  |
| **Testing hierarchy** | |
| **Proposal 1.4: Remind clinical trial teams of the importance of collecting HRQoL data**: Industry should communicate with healthcare providers and clinical teams to educate them on the importance of HRQoL data | **Proposal 2.2: Provide more feedback on the quality and appropriateness of HRQoL data submitted, and any challenges to be addressed in future trials**: CT opinions are usually limited in the review HRQoL data – particularly where this data was rejected. As the reasons for rejection provide an opportunity to learn for industry, we propose include a more detailed commentary on the HRQoL data, regardless of whether this data is accepted or rejected. This would allow to build precedent that can be used to improve clinical trial design, as well as build a virtuous cycle with industry |
| **Proposal 1.5: Clarify the HRQoL score(s) of interest upfront in the stats analysis plan**: To ensure that the most relevant PROs are considered as part of the appraisal, industry should pre-specify which of the PRO summary score or sub-score will be analyzed and which of these is expected to show a benefit to patient or reduce number of PROs to be measured |  |
|  | **Proposal 2.3: Where possible, consider HRQoL data that was not included in a testing hierarchy**: HRQoL data and efficacy data are both central to the value assessment undertaken by HTAs. Currently, the requirement to include both efficacy and HRQoL data in a single endpoint hierarchy is in effect forcing industry to choose between those two endpoints when designing trials – even though these are vastly different types of data. Given the intrinsic differences between HRQoL / efficacy and the importance of assessing both data types, we therefore propose reviewing these data without expecting a single endpoint hierarchy (this assumes they are still secondary endpoints, and so in the pre-defined statistical analysis plan). This would also move HAS closer to the current IQWiG process, which could be a first step towards the EUnetHTA joint clinical appraisal |
|  | **Proposal 2.4: Consider the value of HRQoL data from RWE studies (and, if relevant, develop appropriate guidance on how to obtain, analyse and report RWE data, and clarify the circumstances in which these would be most useful):** We propose firstly that the impact of HRQoL data collected as part of "access précoce" is clarified. Survey results indicated that the majority of HTA experts believed that RWE could compliment HRQoL data as part of the appraisal process. Moreover, IQWiG currently considers RWE where randomized clinical trial data collection is not practical for HRQoL and in some cases (eg. afamelatonide) HAS has stated that RWE HRQoL data contributed to the ASMR determination. We therefore also propose that in cases where HRQoL data were not collected strictly per HAS guidelines, RWE HRQoL data collection could be mandated by HAS and assessed as part of a clearly defined re-evaluation process^1^ |
| **Trial design** | |
| **Proposal 1.6: Minimize the risk of a bias when collecting HRQoL data in open label trials**: When double-blinds are not possible- or importantly if they are no ethical - industry should seek to implement measures which aim to minimize trial bias, such as incorporating partial blinds | **Proposal 2.5: Define specific circumstances where using HRQoL data from open-label trials would be acceptable**: There are cases in which double blinding is not desirable or unethical. Indeed, some open-label studies (eg. extensions) allow for longitudinal analyses which could improve HRQoL data collection by allowing a longer timeframe to be analyzed. Building on the HAS doctrine, which makes allowances for exceptions, we propose to allow data from open-label trials where these cannot be avoided / a rational is provided. As stated earlier, this would require industry to submit strong rational for these open-label trials. Literature suggests no positive bias in PROs collected as part of open-label studies^[[1]](#footnote-2)^, indicating that this proposal would not affect the overall validity of the data considered in the appraisal process. Similarly, HAS should ensure data from innovative trial designs are acceptable (cf. Ministry of Health directive on innovative designs^[[2]](#footnote-3)^) |
|  | **Proposal 2.6: Clarify the guidance for the type of instrument to use**: To ensure that no redundant data is collected, and industry submits consistently high quality HRQoL data for the appraisals, we propose that HAS issues further guidance on instrument requirements and preferences such as translation, specificity for different TAs and patient relevance. This could be the perfect occasion to engage PAGs on the best instruments to use and their relevance to patients |
| **Proposal 1.7: Submit detailed rationales for using HRQoL data from open-label trials**: In cases where double-blinds are not possible – or again if not ethical - industry should clearly communicate in their dossier why this is the case and what mitigation measures have been taken which would make the data acceptable | **Proposal 2.7: Consider the role of novel trial designs and their role in collecting HRQoL data in future studies**: We propose that you consider accepting the role of synthetic arms & historic data as comparators in a select set of exceptional cases. |
| **Data collection** | |
| **Proposal 1.8: Use digital technologies where this could improve the quality of HRQoL data or the patient experience**: With the increasing digital nativity of patients in the clinic, industry should seek to design new, easier, and more streamlined processes for patients to report outcomes. Note: these technologies should not come at the expense of patients not comfortable with digital technologies | **Proposal 2.8: Clarify the relative importance of HRQoL vs other clinical parameters such as efficacy**: As introduced earlier, patient perception is one of the four key areas which should determine acceptability of the data. During our AdBoards, as well as the interviews and survey we conducted, the perception that HRQoL data is in secondary importance to safety and efficacy was brought up. As our first proposal, we suggest clarifying the relative importance that you attach to HRQoL data, compared to efficacy and safety in your assessment |
|  | **Proposal 2.9: Consider differential recommendations for products where they have a similar efficacy but different impacts on patient HRQoL**: We propose that you explore differential product recommendations in clinical guidelines where meaningful differences in HRQoL data exists alongside the traditional appraisal process (or where HRQoL data exists for one product but not for others) |
| **Proposal 1.9: Simplify HRQoL instruments used by reducing their length and improve relevance to patients**: It will therefore be crucial for industry to work with patients and PAGs to both (1) reduce the length of the questionnaires, (2) simplify the forms and (3) ensure that the questions asked are relevant to patient | **Proposal 2.10: Support the definition of a Europe-wide threshold for missing data as part of EUnetHTA or discuss methods that will allow to decrease the impact of missing data in industry submissions**: Missing data is largely an issue with industry needs to address (eg. using simpler, patient-relevant, validated & translated instruments), but HAS could nonetheless investigate a common threshold for missing data with other EU agencies ahead of EUnetHTA, and communicate this clearly to industry to set the bar |
| **Proposal 1.10: Thoughtfully choose appropriately validated instruments used in trials; and clearly report on these:** The doctrine has several requirements on the validation and language of instruments used. Industry should ensure these methodological requirements are met |  |

**Supplemental Table 1.** Table of 10 proposals to industry and 10 proposals to HAS to improve HRQoL data adoption in France, framed around the four key areas of work: patient perception, testing hierarchy, trial design and data collection.

1. Efficace et. al., ”Impact of Blinding on Patient-Reported Outcome Differences Between Treatment Arms in Cancer Randomized Controlled Trials”, 2021 [↑](#footnote-ref-2)
2. http://www.nile-consulting.eu/drop/21104-Lettre_de_saisine_OV-HAS.pdf [↑](#footnote-ref-3)
